# The complete mitochondrial genome of *Stolephorus commersonii*

**DOI:** 10.1080/23802359.2017.1372707

**Published:** 2017-08-30

**Authors:** Liyan Qu, Chunyan Ma, Hongyu Ma, Yao Dong, Wei Wang, Guijing Ren, Lingbo Ma

**Affiliations:** a Key Laboratory of East China Sea and Oceanic Fishery Resources Exploitation, Ministry of Agriculture, East China Sea Fisheries Research Institute, Chinese Academy of Fishery Sciences, Shanghai, China;; b College of Fisheries and Life Sciences, Shanghai Ocean University, Shanghai, China

**Keywords:** *Stolephorus commersonii*, mitochondrial DNA, mitogenome

## Abstract

In this study, the complete mitochondrial genome of *Stolephorus commersonii* is determined. It is 16,734 bp in length and consists of 13 protein-coding genes, 22 transfer RNA (tRNA) genes, two ribosomal RNA (rRNA) genes, and a control region. Phylogenetic tree was constructed based on the complete mitogenome of *S. commersonii* and closely related 17 other species to assess its phylogenic relationship and evolution. The findings of the study will contribute to the phylogenetic classification and the genetic conservation management of *S. commersonii.*

*Stolephorus commersonii* is an anadromous fish in the Engraulidae family. It wildly distributes in the India and Pacific Ocean. In China, it locates in the southeast coastal areas (Lacepè 1803; Ni and Wu 2006). In the present study, the complete mitochondrial DNA sequence of *S. commersonii* has been determined.

The specimen of *S. commersonii* was collected from Fuqing, Fujian, China (25°50′2″N; 119°27′35″E) in December 2009. Genomic DNA was extracted from muscle tissue using Animal Genomic DNA Extraction Kit (TIANGEN, Beijing, China) according to the manufacturer's recommended protocol and was stored in East China Sea Fisheries Research Institute, China. The transcriptome of *S. commersonii* has been sequenced by the Roche 454 Genome Sequencer FLX System (Ma et al. 2013). The complete mitochondrial genome of *S. commersonii* was 16,734 bp in length (GenBank accession no. KX753639). The total base composition of its mitogenome is 29.13% for A, 27.20% for T, 26.80% for C, and 16.88% for G. The overall A + T content of the mitochondrial genome is 56.33%. The A + T content of control region and protein-coding genes is 64.57% and 56.55%.

The total length of 13 protein-coding genes was 11,418 bp and the A + T and G + C content was 56.55% and 43.45%, respectively. The lengths of two ribosomal RNA genes (12S rRNA and 16S rRNA), were 950bp and 1703 bp, respectively. The control region of *S. commersonii* is 1099 bp in length. The base content of two rRNA genes was 32.55% for A, 20.59% for G, 21.69% for T, and 25.17% for C. As described in other fishes (Brown et al. 1986; Lee et al. 1995; Hurst et al. 1999), the D-Loop is possessed of the typical tripartite regions: extended termination associated sequence (ETAS) domain, central conserved sequence block (CSB-D, E, F) domain, and conserved sequence block (CSB-1, 2, 3) domain.

In order to assess its phylogenic relationship and evolution, the tree was constructed with significant bootstrap supports based on the maximum-likelihood method (Figure 1). The tree topologies suggested that *S. commersonii* had close relationship with *S. chinensis* and *S. waitei*. The Engraulis is closest to the Stolephorus under the family of Engraulidae in this study. This study will contribute to the phylogenetic classification and the genetic conservation management of *S. commersonii.*

**Figure 1.**
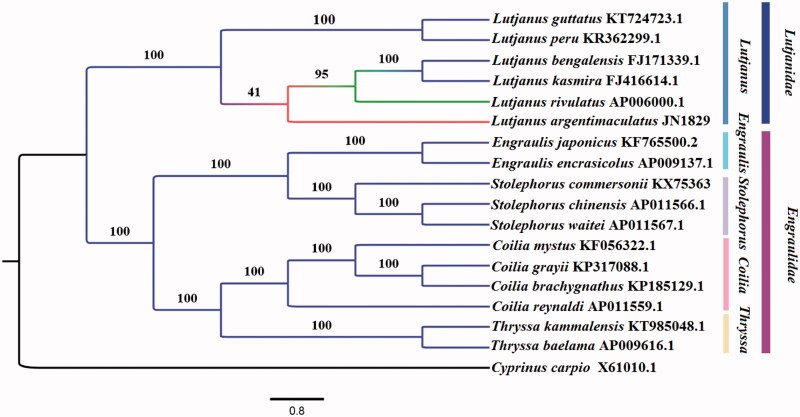
Maximum-likelihood tree of complete mitogenomes of *S. commersonii* and 17 other species with NCBI accession number.
